# Hypoxia Modulates Effects of Fatty Acids on NES2Y Human Pancreatic β-cells

**DOI:** 10.3390/ijms20143441

**Published:** 2019-07-12

**Authors:** Jan Šrámek, Vlasta Němcová-Fürstová, Jan Polák, Jan Kovář

**Affiliations:** 1 Department of Biochemistry, Cell and Molecular Biology & Center for Research of Diabetes, Metabolism and Nutrition, Third Faculty of Medicine, Charles University, Ruská 87, 110 00 Prague, Czech Republic; 2 Department of Pathophysiology, Third Faculty of Medicine, Charles University, Ruská 87, CZ-100 00 Prague, Czech Republic

**Keywords:** fatty acids, pancreatic β-cells, hypoxia, apoptosis, ER stress, caspases, fatty acid transporters, hypoxia-inducible factor 1α, NES2Y

## Abstract

Saturated fatty acids (FAs) induce apoptosis in the human pancreatic NES2Y β-cell line while unsaturated FAs have nearly no detrimental effect. Moreover, unsaturated FAs are capable of inhibiting the pro-apoptotic effect of saturated FAs. Hypoxia is also known to have deleterious effects on β-cells function and viability. In the present study, we have tested the modulatory effect of hypoxia on the effect of FAs on the growth and viability of the human pancreatic NES2Y β-cells. This study represents the first study testing hypoxia effect on effects of FAs in pancreatic β-cells as well as in other cell types. We showed that hypoxia increased the pro-apoptotic effect of saturated stearic acid (SA). Endoplasmic reticulum stress signaling seemed to be involved while redistribution of FA transporters fatty acid translocase/cluster of differentiation 36 (FAT/CD36) and fatty acid-binding protein (FABP) do not seem to be involved in this effect. Hypoxia also strongly decreased the protective effect of unsaturated oleic acid (OA) against the pro-apoptotic effect of SA. Thus, in the presence of hypoxia, OA was unable to save SA-treated β-cells from apoptosis induction. Hypoxia itself had only a weak detrimental effect on NES2Y cells. Our data suggest that hypoxia could represent an important factor in pancreatic β-cell death induced and regulated by FAs and thus in the development of type 2 diabetes mellitus.

## 1. Introduction

It was suggested that increased levels of fatty acids (FAs) in the blood are factors contributing to the failure and death of pancreatic β-cells. Therefore, it can contribute to the development of type 2 diabetes mellitus (T2DM) [1,2,3,4]. Our previous studies as well as studies of others have shown that the effect of FAs on the function and viability of pancreatic β-cells depends on the level of their saturation. Saturated FAs, i.e., palmitic or more efficiently stearic acid (SA), have a detrimental effect on β-cells whereas unsaturated FAs, i.e., oleic acid (OA) or palmitoleic acid, are well tolerated and are even capable of inhibiting the pro-apoptotic effect of saturated FAs [1,2,3,4].

In the last decade it was suggested that also hypoxia represents a factor contributing to pancreatic β-cell death in T2DM. Direct detrimental effect of hypoxia on pancreatic β-cell function and viability was documented in rodents [5,6,7]. The role of hypoxia in β-cell apoptosis can be supported by the documented presence of hypoxia in vivo in the islets of animal models of T2DM [8,9]. Hypoxia is probably developed in the pancreas mainly due to obstructive sleep apnea syndrome (SAS). SAS was found to affect as much as 30%–40% of obese subjects [10] and correlation between SAS and T2DM development was proved [11,12,13].

Molecular mechanisms of apoptosis induction by saturated FAs and inhibition of this induction by unsaturated FAs as well as molecular mechanisms underlying the deleterious effect of hypoxia in β-cells remain unclear [14,15]. However, beside the activation of caspases leading to poly ADP-ribose polymerase (PARP) cleavage and apoptosis execution, endoplasmic reticulum (ER) stress signaling seems to be involved [4,6].

ER stress was demonstrated to result in the activation of three signaling pathways: (1) Inositol-requiring protein 1α (IRE1α) pathway, (2) protein kinase RNA (PKR)-like ER kinase (PERK) pathway, and (3) activating transcription factor 6 (ATF6) pathway. Activation of IRE1α leads to JNK (c-Jun N-terminal kinase) activation via phosphorylation. Activated PERK results in the inhibition of protein translation via eIF2α (eukaryotic initiation factor 2α) phosphorylation. ATF6 after its activation by cleavage translocates to the nucleus where it functions as a transcription factor. These pathways primarily participate in the restoration of ER homeostasis, e.g., by increasing the expression of chaperones, such as immunoglobulin heavy chain-binding protein (BiP) [16]. However, if this response fails, caspases are activated and apoptosis is induced by mechanisms that are still not completely understood. A suggested mediator here is a transcription factor known as the CCAAT-enhancer-binding protein (C/EBP) homologous protein (CHOP) [17]. BiP and CHOP proteins are therefore considered to be the main markers of ER stress (reviewed in [14]).

It was documented in cardiac myocytes that strong hypoxia induces the redistribution of two FAs transporters, fatty acid translocase/cluster of differentiation 36 (FAT/CD36), and fatty acid-binding protein (FABP), from the intracellular pool to the plasma membrane. It leads to increased FAs accumulation [18]. Both transporters were documented to be present in pancreatic β-cells [19,20].

In in vitro studies, various factors influencing β-cell viability, e.g., increased glucose level, increased FA level, hypoxia, are usually studied separately with the exception concerning the interplay of high glucose and high FA concentration. However, in the case of in vivo systems, these different factors interplay. In the present study, we tested the modulatory effect of hypoxia on the effects of FAs on growth and viability of the human pancreatic β-cells NES2Y in an attempt to mimic the milieu present in the human body of obese individuals. This seems to be the first study testing hypoxia effect on effects of FAs in pancreatic β-cells as well as in other cell types. We documented that hypoxia increased the pro-apoptotic effect of SA. ER stress signaling seemed to be involved while redistribution of FA transporters FAT/CD36 and FABP do not seem to be involved in this effect. Hypoxia also strongly decreased the protective effect of OA against the pro-apoptotic effect of SA. Thus, OA in the presence of hypoxia was unable to save SA-treated β-cells from apoptosis induction. Hypoxia itself had only a weak detrimental effect on NES2Y cells. These results suggest that hypoxia could represent an important factor in pancreatic β-cell death induced and regulated by FAs and thus in the development of T2DM.

## 2. Results

### 2.1. Effect of Hypoxia and Fatty Acids on HIF1α Expression

**Figure 1:**
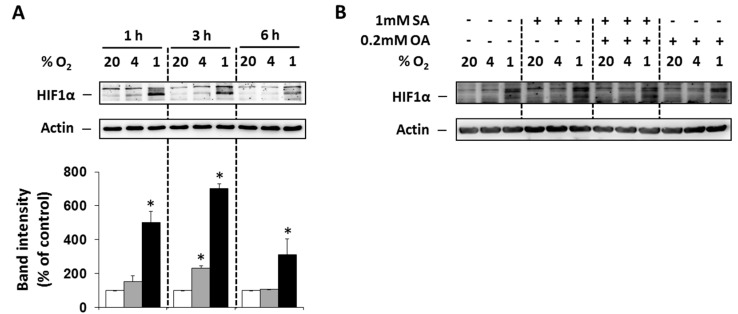
(**A**) Effect of hypoxia (see “Materials and Methods”) on the level of HIF1α within 6 h of the treatment in NES2Y cells. (**B**) Effect of hypoxia applied with 1 mM stearic acid (SA), 1 mM SA plus 0.2 mM oleic acid (OA), and 0.2 mM OA (see “Materials and Methods”) on the level of HIF1α 3 h after the treatment in NES2Y cells. Level of HIF1α was determined using western blot analysis employing relevant antibody (see “Materials and Methods”). The actin level was used to confirm equal protein loading. The data shown were obtained in one representative experiment from at least three independent experiments. Densitometric analysis concerning data (**A**) is also shown. * *p* < 0.05 when comparing the effect of a particular hypoxia with external normoxia (20% O_2_, control).

To prove that selected O_2_ concentrations (4% and 1%) lead to hypoxia in NES2Y cells, the level of hypoxia-inducible factor 1α (HIF1α) was analyzed. Moderate (4% O_2_) and particularly strong (1% O_2_) hypoxia increased expression of HIF1α within 6 h of treatment with the maximum effect at 3 h compared to control cells (20% O_2_). The effect of strong hypoxia was markedly stronger than the effect of moderate hypoxia (Figure 1A). We also tested the effect of FAs under hypoxia on HIF1α expression 3 h after the treatment. Application of the 1 mM stearic acid (SA), 1 mM SA plus 0.2 mM oleic acid (OA), and 0.2 mM OA led only to minimal changes in HIF1α expression (Figure 1B).

### 2.2. Modulation of the Effects of Fatty Acids on Cell Growth and Viability by Hypoxia

**Figure 2:**
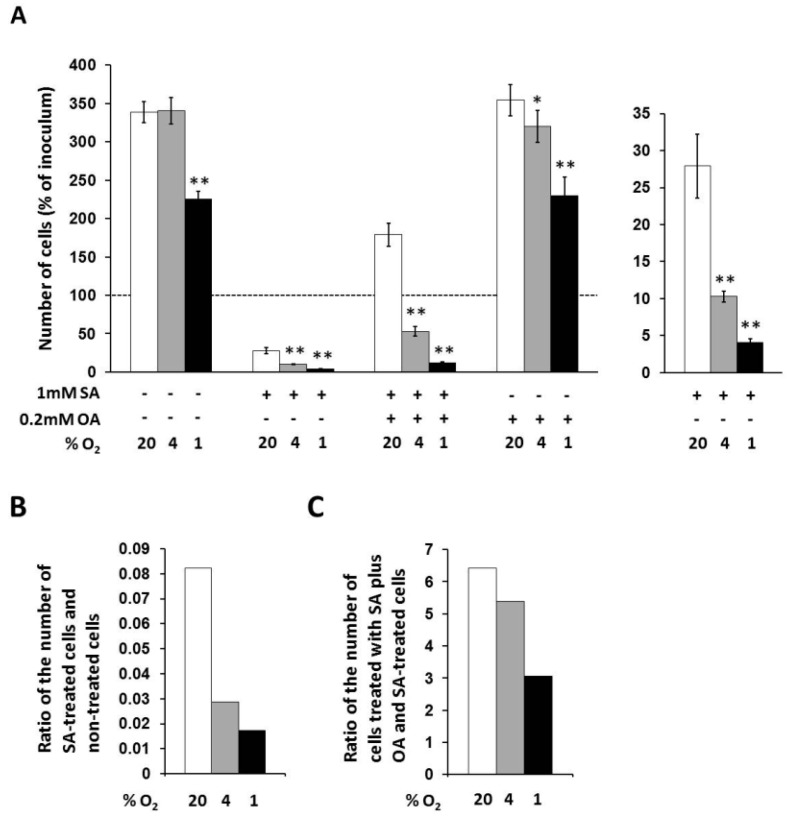
(**A**) Effects of hypoxia applied alone and simultaneously with 1 mM stearic acid (SA), 1 mM SA plus 0.2 mM oleic acid (OA), and 0.2 mM OA (see “Materials and Methods”) on cell growth and viability of NES2Y cells. (**B**) Effect of hypoxia on cell growth and viability of NES2Y cells treated with SA when compared to cells without fatty acid (ratio of the number of SA-treated cells and non-treated cells). (**C**) Effect of hypoxia on cell growth and viability of NES2Y cells treated with SA plus OA when compared to SA-treated cells (ration of the number of cells treated with SA plus OA and SA-treated cells). The number of living cells was determined after 48 h of incubation in the presence of hypoxia (4% and 1% O_2_) or under control conditions (20% O_2_). Each column represents the mean of three experimental values ± SEM. * *p* < 0.05, ** *p* < 0.001 when comparing the effect of a particular hypoxia with external normoxia. The dotted line represents the number of cells of inoculum.

In non-treated cells, only strong hypoxia decreased the number of living cells to approximately 65% of the number of cells under external normoxia within 48 h of incubation (Figure 2A).

Under control conditions (20% O_2_), 1 mM stearic acid (SA) decreased the number of living NES2Y cells approximately to 28% of the number of non-treated cells, i.e., significantly below the number of cells of inoculum, within 48 h of incubation. Moderate hypoxia (4% O_2_) produced a further significant decrease of the number of cells treated with 1 mM SA within the same incubation period. The number of living cells under moderated hypoxia was about 10% of the number of cells under normoxia. The ratio of the number of SA-treated cells and non-treated cells was decreased from 0.082 (normoxia) to 0.029 by moderate hypoxia. Strong hypoxia (1% O_2_) decreased the number of cells treated with SA more than moderate hypoxia. The number of living cells under strong hypoxia represented approximately 4% of the number of cells under normoxia. The ratio of the number of SA-treated cells and non-treated cells was decreased from 0.082 (normoxia) to 0.017 by strong hypoxia (see Figure 2A,B).

Under control conditions (20% O_2_), 0.2 mM oleic acid (OA) applied together with 1 mM SA increased the number of living cells approximately 6.5-fold compared to the number of cells treated with SA only, i.e., significantly over the number of cells of inoculum, within 48 h of incubation. Moderate hypoxia significantly decreased this enhancing effect of OA. The number of living cells under moderate hypoxia was increased due to OA co-application with SA only 5.4-fold compared to the number of cells treated with SA only, i.e., significantly below the number of cells of inoculum. Strong hypoxia decreased the enhancing effect of OA more than moderate hypoxia. The number of living cells under strong hypoxia was increased after OA co-application only 3.1-fold compared to the number of cells treated with SA only (see Figure 2A,C).

OA at a concentration of 0.2 mM had no effect on the number of living cells under control conditions (20% O_2_) within 48 h of incubation. Hypoxia seemed to have a similar effect on OA-treated cells like on non-treated cells (Figure 2A).

### 2.3. Modulation of the Effect of Fatty Acids on the Activation of Caspases by Hypoxia

**Figure 3:**
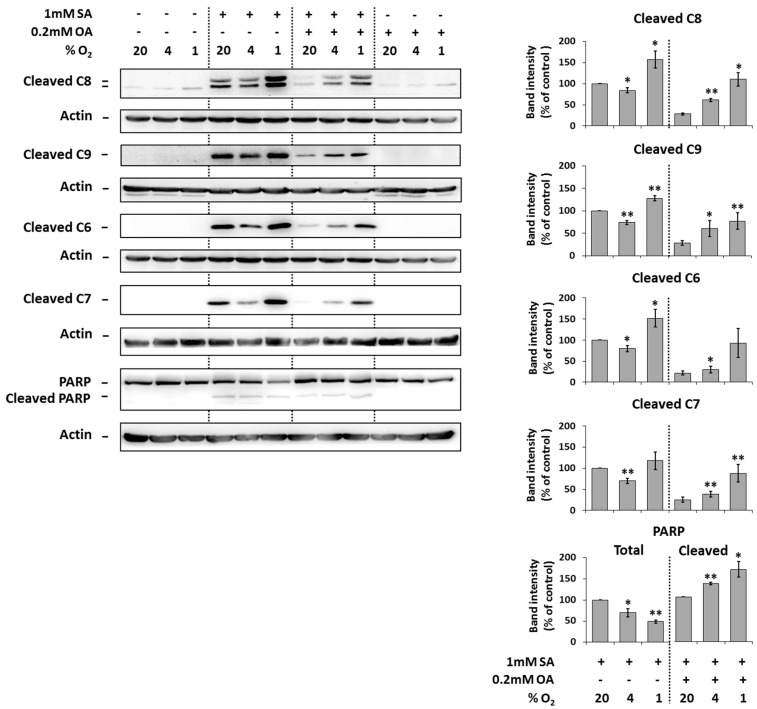
Effect of hypoxia (see “Materials and Methods”) applied alone and simultaneously with 1 mM stearic acid (SA), 1 mM SA plus 0.2 mM oleic acid (OA), and 0.2 mM OA (see “Materials and Methods”) on the activation of caspase-8 (C8), caspase-9 (C9), caspase-6 (C6), caspase-7 (C7) assessed by the level of cleaved caspases and on the level of PARP cleavage in NES2Y cells. After 18 h of incubation, levels of individual proteins were determined using western blot analysis employing relevant antibodies (see “Materials and Methods”). The actin level was used to confirm equal protein loading. The data shown were obtained in one representative experiment from at least three independent experiments. Densitometric analysis of key data from western blotting is also shown. Each column represents the mean of three experimental values ± SEM. * *p* < 0.05, ** *p* < 0.01 when comparing with the effect of 1 mM SA (or 1 mM SA + 0.2 mM OA in the case of cleaved PARP) under 20% O_2_ (control).

Under the control conditions (20% O_2_), the application of 1 mM SA alone resulted in significant activation (cleavage) of initiator caspase-8, -9 as well as executioner caspase -6, -7 and the cleavage of caspase substrate PARP in NES2Y cells compared to non-treated cells after 18 h of incubation. Moderate hypoxia (4% O_2_) seemed to slightly decrease the level of cleaved caspases but not of the PARP in cells treated with SA. Strong hypoxia (1% O_2_) increased the level of caspases and PARP cleavage (Figure 3).

The application of 0.2 mM OA together with 1 mM SA (20% O_2_) decreased the cleavage of caspases and PARP due to SA application within 18 h of incubation. Moderate as well as strong hypoxia counteracted the protection effect of OA against cleavage of caspases and PARP due to SA application (Figure 3).

The application of OA alone at a concentration of 0.2 mM did not result in caspase activation and PARP cleavage within 18 h of incubation. Hypoxia did not affect it. Hypoxia itself had nearly no effect on caspase activation and PARP cleavage (Figure 3).

### 2.4. Modulation of the Effect of Fatty Acids on ER Stress Signaling by Hypoxia

**Figure 4:**
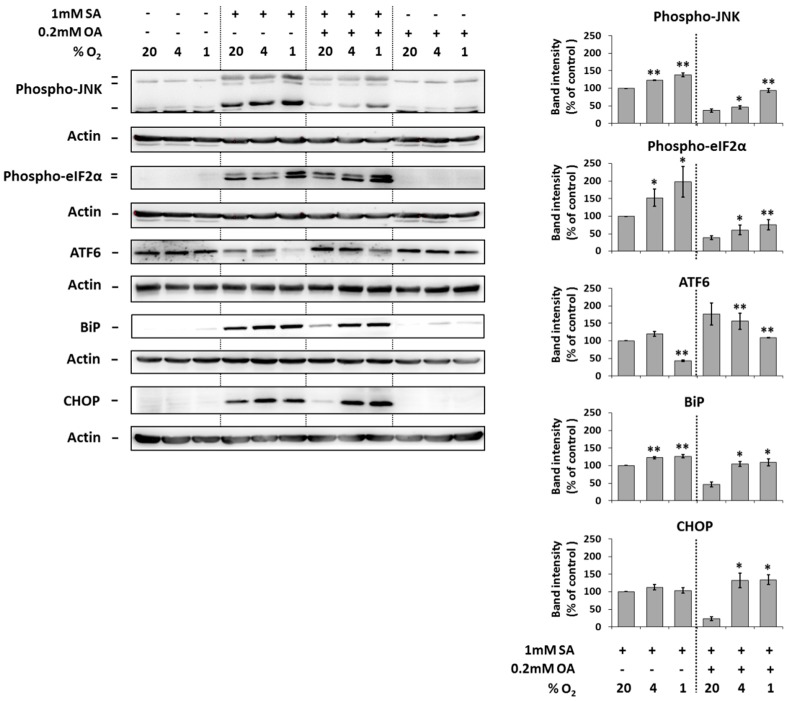
Effect of hypoxia (see “Materials and Methods”) applied alone and simultaneously with 1 mM stearic acid (SA), 1 mM SA plus 0.2 mM oleic acid (OA), and 0.2 mM OA (see “Materials and Methods”) on the level of phospho-JNK, phospho-eIF2α, ATF6, BiP, and CHOP in NES2Y cells. After 18 h of incubation, levels of individual proteins were determined using western blot analysis employing relevant antibodies (see “Materials and Methods”). The actin level was used to confirm equal protein loading. The data shown were obtained in one representative experiment from at least three independent experiments. Densitometric analysis of key data from western blotting is also shown. Each column represents the mean of three experimental values ± SEM. * *p* < 0.05, ** *p* < 0.01 when comparing with the effect of 1 mM SA under 20% O_2_ (control).

The application of 1 mM SA alone (20% O_2_) resulted in a significant increase of the phosphorylation of JNK and eIF2α, the cleavage of ATF6 and increase of the expression of endoplasmic reticulum stress markers BiP and CHOP in NES2Y cells after 18 h of incubation. Hypoxia seemed to potentiate these effects of SA probably with the exception of the effect on CHOP expression (Figure 4).

The application of 0.2 mM OA together with 1 mM SA (20% O_2_) significantly decreased the effects of SA on the phosphorylation of JNK and eIF2α, the cleavage of ATF6 and the expression of BiP and CHOP after 18 h of incubation. Moderate and strong hypoxia counteracted this OA inhibitory effect on effects of SA (Figure 4).

The application of 0.2 mM OA alone (20% O_2_) had no effect on the phosphorylation of JNK and eIF2α, cleavage of ATF6, and the expression of BiP as well as CHOP. Hypoxia did not significantly change it (Figure 4).

Hypoxia itself had nearly no effect on the expression, cleavage, or phosphorylation of tested proteins (Figure 4).

### 2.5. Effect of Hypoxia on Redistribution of FA Transporters FAT/CD36 and FABP

**Figure 5:**
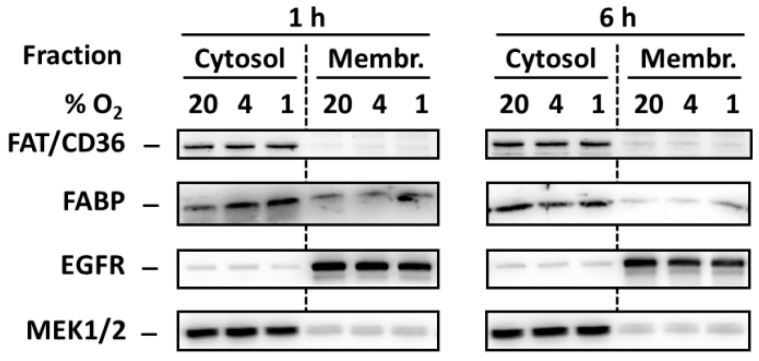
Effect of hypoxia (see “Materials and Methods”) on the level of FAT/CD36 and FABP 1 and 6 h after the treatment in cytosolic and membrane-enriched fraction of NES2Y cells. Levels of the tested proteins were determined using western blot analysis employing relevant antibodies (see “Materials and Methods”). Equal protein load was checked after blotting by staining with Ponceau S solution. The level of EGFR (membrane protein) and MEK1/2 (cytosolic protein) were used to confirm that the preparation of fractions was successful. The data shown were obtained in one representative experiment from at least three independent experiments.

In order to test whether hypoxia induces redistribution of FA transporters FAT/CD36 and FABP from the intracellular pool to the plasma membrane we analyzed levels of these transporters in cytosol and membrane-enriched fraction. However, hypoxia did not change significantly the levels of both transporters in both fractions (Figure 5).

## 3. Discussion

Previous studies have demonstrated that saturated fatty acids (FAs), e.g., stearic acid (SA) and palmitic acid, induce apoptosis in the human pancreatic β-cell line NES2Y while unsaturated FAs, e.g., oleic acid (OA) and palmitoleic acid, have a weak detrimental effect only at high concentrations. Moreover, unsaturated FAs are capable of inhibiting the pro-apoptotic effect of saturated FAs ([1,2,3,4], see also Figure 2A). Besides saturated FAs, hypoxia was also demonstrated to have a deleterious effect on the function and viability of β-cells [6,7,8,9]. In this study, we tested the modulation of effects of FAs on the growth and viability of the human pancreatic NES2Y β-cells by hypoxia. To our knowledge, this is the first study testing hypoxia effect on effects of FAs in pancreatic β-cells as well as in other cell types.

We have documented that moderate (4% O_2_) as well as strong (1% O_2_) hypoxia significantly increased the pro-apoptotic effect of saturated SA in NES2Y β-cells. The effect of strong hypoxia was significantly stronger then the effect of moderate hypoxia (see Figure 2A,B and Figure 3). Concerning these data, it seems that a weak deleterious effect of hypoxia, when combined with other pro-apoptotic factor(s), may represent a decisive element leading to pancreatic β-cell death. Potentiation of the cytotoxic effect of various agents due to hypoxia was published in other cell types [21,22]. We have also shown that hypoxia strongly decreased the protective effect of unsaturated OA against the pro-apoptotic effect of saturated SA. Thus, OA was unable to block apoptosis induction in SA-treated β-cells. The effect of strong hypoxia was again stronger than the effect of moderate hypoxia (see Figure 2A,C and Figure 3). Concerning these results, it seems that hypoxia can also inhibit anti-apoptotic effects in pancreatic β-cells. However, there are no studies, to our knowledge, published considering this issue. Similar effect of hypoxia on the effects of FAs concerning cell growth and viability was also obtained using rat INS-1E cells (see Figure S1). Regarding our results about the effect of hypoxia applied alone, we have found, that only strong hypoxia (1% O_2_) has a significant effect on cell growth of NES2Y cells (see Figure 2A). Interestingly, in rodent β-cell lines (INS-1, MIN6), moderate or strong hypoxia caused much stronger deleterious effects [5,6,23,24].

Concerning hypoxia detrimental effects in NES2Y cells it seems that caspases (caspase-9, -8, -7 and -6) and PARP reflect them since FAs-induced cleavage of these proteins was increased under hypoxic conditions (see Figure 3). We have also preliminary data suggesting that caspase-2 is activated in response to hypoxia. A possible involvement of caspase-9, -8 and -3 in hypoxia effect is mentioned in literature [5,6,23,24] but this is the first study suggesting possible involvement of caspase-6 and -7 as mediators of hypoxia deleterious effects in pancreatic β-cells. Interestingly, moderate hypoxia decreased saturated SA-induced cleavage of caspases (see Figure 3) and it was not followed by an increase of the number of living cells. On the contrary, decrease of the number of living cells was observed (see Figure 2A). It can represent some other mechanism(s) which is involved.

We have also shown that hypoxia increases FAs-induced activation of IRE1α, PERK, and ATF6 ER stress pathways and expression of the main ER stress markers BiP and CHOP (see Figure 4). These results as well as the results of some other authors [6,25] suggest that ER stress signaling could mediate detrimental effects of hypoxia in pancreatic β-cells. FA-induced ER stress could be potentiated by hypoxia in β-cell probably since the appropriate folding of proinsulin in the ER involves the formation of three disulphide bonds, a process that requires molecular oxygen [26]. Therefore, a lack of molecular oxygen leads to the accumulation of unfolded proinsulin and other proteins. It can decrease β-cell function and, together with FAs, contributes to the disruption of ER homeostasis leading to pancreatic β-cell death. ER stress signaling was generally considered to be upstream from caspase activation [17]. Therefore, hypoxia intervention into molecular mechanisms of apoptosis induction by saturated FAs and into mechanisms of inhibition of this induction by unsaturated FAs should be located within or upstream of ER stress pathways.

It was documented in cardiac myocytes that strong hypoxia induces the redistribution of two FAs transporters, FAT/CD36 and FABP, from the intracellular pool to the plasma membranes leading to increased FAs accumulation [18]. Both transporters were documented to be present in pancreatic β-cells [19,20]. One can speculate that such hypoxia-induced redistribution leading to FAs uptake can also occur in β-cells and that this effect may also represent the mechanism by which hypoxia potentiate FAs-induced apoptosis induction in pancreatic β-cells. Moreover, increased uptake of FAs in cells under hypoxia conditions may result in a situation when OA (which is generally protective [2,4] but can have at higher concentrations also detrimental effects on β-cells [27,28]) has no more cytoprotective effect in the β-cell but, on the contrary, has pro-apoptotic effect. Similarly, OA may not be already able to inhibit pro-apoptotic effects of SA whose concentrations were increased in cells due to hypoxia-induced uptake. However, we did not found redistribution of both FA transporters, mentioned above, due to hypoxia in NES2Y pancreatic β-cells (see Figure 5).

To conclude, we demonstrated that hypoxia increased pro-apoptotic effect of SA in the human pancreatic NES2Y β-cells. ER stress signaling could be involved while redistribution of FA transporters FAT/CD36 and FABP do not seem to participate here. Hypoxia strongly decreased the protective effect of OA against the pro-apoptotic effect of SA. Thus, in the presence of hypoxia, OA was unable to save SA-treated β-cells from apoptosis induction. Hypoxia itself had only a weak detrimental effect on NES2Y cells. Our data suggest that hypoxia could represent an important factor in pancreatic β-cell death induced and regulated by FAs and, thus, in the development of T2DM. These data also indicate that it may be necessary to reassess our recent knowledge concerning the effects and mainly the importance of the individual factors involved in T2DM development.

## 4. Materials and Methods

### 4.1. Materials

All chemicals were sourced from Sigma-Aldrich (St. Louis, MO, USA), unless otherwise stated. For the western blot analysis, the following primary and secondary antibodies were used: Anti-cleaved caspase-6 (#9761), anti-cleaved caspase-7 (#9491), anti-cleaved caspase-8 (#9496), anti-cleaved caspase-9 (#9505), anti-PARP (#9542), anti-ATF6 (#65880), anti-BiP (#3177), anti-CHOP (#2895), anti-EGFR (#4267), and anti-MEK (#8727) from Cell Signaling Technology (Danvers, MA, USA). Anti-H-FABP (ab45966) and anti-CD36 (ab133625) from Abcam (Cambridge, UK), and anti-actin (clone AC-40).

### 4.2. Cells and Culture Conditions

The human pancreatic β-cell line NES2Y [1,29] was used. Cells were routinely maintained in an RPMI 1640 based culture medium [30]. In experiments, cells were seeded according to the experimental protocols described below on 50 mm culture dishes or on 24-well plates. Both plastic had a bottom made from a fluorocarbon membrane (Lumox, Sarstedt AG & Co, Nümbrecht, Germany) enabling direct diffusion of gasses (O_2_, CO_2_) to the pericellular space as previously used [31,32]. In experiments, a defined serum-free medium [33] supplemented with fatty acids (FAs), (1 mM stearic acid (SA), a combination of 1 mM SA and 0.2 mM oleic acid (OA), or 0.2 mM OA alone) bound to a 2% FA-free bovine serum albumin (BSA) was used [1]. Stock solutions containing FA(s) bound to 10% BSA in a serum-free medium were prepared as described previously [1] and diluted to the required concentration of FA and BSA prior to experiments. FA/BSA molar ratios used in the experiments were lower than the ratios known to exceed the binding capacity of BSA [34].

Our previous studies showed that SA, at a concentration of 1 mM, induces endoplasmic reticulum stress and apoptosis in most NES2Y cells within 24 h of application [4,35,36,37]. Therefore, all assessments were performed within 24 h after FAs application except for the assessment of cell growth and viability. A 1 mM concentration of SA was used to simulate an elevated level of SA in the blood [38,39]. A 0.2 mM concentration of OA was used since this was the lowest concentration sufficient to inhibit the detrimental effects of SA [1].

### 4.3. Assessment of the Effect of Hypoxia on Cell Growth and Viability

Cells were seeded at a concentration of 9 × 10^4^ cells/100 µL of culture media into the wells of a 24-well plate. After a 24-h pre-incubation period (allowing cells to attach), the culture medium was replaced with a serum-free medium containing 2% BSA with or without FA(s) (SA, a combination of SA and OA, or OA alone) at required concentrations. Cells were placed inside a standard incubation cabinet providing a 20% oxygen level or inside chambers of a specific incubation cabinet [31] in which oxygen levels were 4% or 1%. A standard incubation cabinet (20% oxygen level) represents external normoxia of the environment. It is commonly used in in vitro systems by other authors [5,6,23]. It provides sufficient oxygen concentration for normal β-cells function. It was suggested that physiological oxygen concentration in pancreatic islets is around 6.3% [40,41]. Therefore, incubation in a specific incubation cabinet providing 4% oxygen concentration represents moderate hypoxia for pancreatic β-cells. Incubation in a 1% oxygen concentration represents strong hypoxia. To prove that these oxygen concentrations lead to hypoxia in NES2Y cells the level of the main marker of hypoxia HIF1α was analyzed (Figure 1). Hypoxia concentrations used in this study are commonly used by other authors [5,6,23]. After 48 h of incubation, the number of living cells was determined using a haemocytometer counting system, after staining with trypan blue.

### 4.4. Western Blot Analysis

Cells (approximately 1 × 10^6^ cells per sample) were seeded and after a 24-h pre-incubation period (allowing cells to attach), the culture medium was replaced with a serum-free medium containing 2% BSA with or without FA(s) (SA, a combination of SA and OA, or OA alone) at required concentrations. Cells were placed inside a standard incubation cabinet (20% oxygen level, external normoxia) or inside chambers of a specific incubation cabinet [31] in which oxygen levels were 4% (moderate hypoxia) or 1% (strong hypoxia). After relevant time of incubation, cells were harvested and the western blot analysis was performed as described previously [4]. All primary antibodies were used in a 1:1000 dilution. The chemiluminescent signal was detected using a Carestream Gel Logic 4000 PRO Imaging System equipped with Carestream Molecular Imaging Software (Carestream Health, New Haven, CT, USA), which was used for image acquisition. Image Master^TM^ 2D Platinum 6.0 software (GE Healthcare, Uppsala, Sweden) was used to obtain data for densitometric analysis.

### 4.5. Preparation of Membrane-Enriched Fractions

Cells were seeded and treated with hypoxia as described in “western blot analysis”. Cells were harvested after 1 and 6 h of incubation. Mem-PER Plus Membrane Protein Extraction kit (ThermoFisher Scientific, Waltham, MA, USA) was used according to manufacturer’s instructions to isolate cytosolic proteins and membrane together with membrane-associated proteins, resulting in production of a highly enriched cytosol and membrane samples (fractions). Prior to western blot analysis, the quality of all membrane sample preparations, relative to selectivity and purity, was tested by comparison of the content of MEK1/2 and EGFR proteins, typical for their cellular localization, in the cytosolic and membrane fractions.

### 4.6. Statistical Analysis

Normality of data was tested using Shapiro–Wilk test. The statistical significance of observed differences was determined using the Student’s *t*-test. *p* < 0.05 was considered as statistically significant.

## Supplementary Materials

Supplementary materials can be found at https://www.mdpi.com/1422-0067/20/14/3441/s1.

## Abbreviations

ATF6	Activating transcription factor 6
BiP	Immunoglobulin heavy chain-binding protein
CHOP	CCAAT-enhancer-binding protein (C/EBP) homologous protein
ER	Endoplasmic reticulum
FABP	Fatty acid-binding protein
FAT/CD36	Fatty acid translocase/cluster of differentiation 36
FAs	Fatty acids
HIF1α	Hypoxia-inducible factor 1α
IRE1α	Inositol-requiring protein 1α
JNK	c-Jun N-terminal kinase
PARP	Poly ADP-ribose polymerase
PERK	Protein kinase RNA (PKR)-like ER kinase
SA	Stearic acid
SAS	Sleep apnea syndrome
T2DM	Type 2 diabetes mellitus
OA	Oleic acid
