# Supplementary material for: Hypoxia Modulates Effects of Fatty Acids on NES2Y Human Pancreatic β-cells
Source: Int J Mol Sci. 2019 Jul 12;20(14):3441. doi: 10.3390/ijms20143441 (PMC6678120; doi:10.3390/ijms20143441)
Supplement: Supplementary file 1 [file ijms-20-03441-s001.pdf]

SUPPLEMENTARY MATERIALS

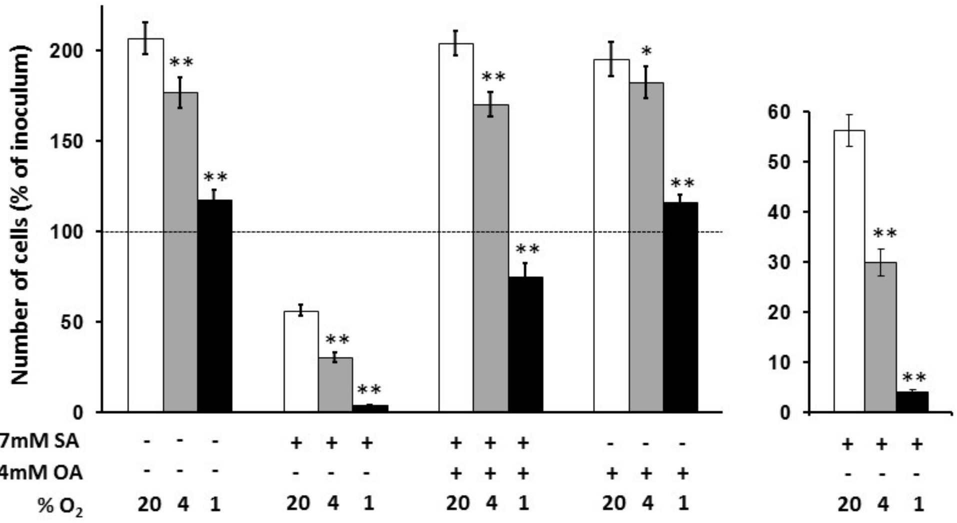

Figure S1. Effects of hypoxia applied alone and simultaneously with 0.7 mM stearic acid (SA), 0.4 mM SA plus 0.4 mM oleic acid (OA), and 0.4 mM OA on cell growth and viability of rat INS-1E cells. The number of living cells was determined after 48 hours of incubation in the presence of hypoxia (4 % and 1 % O_2) or under control conditions (20 % O_2). Each column represents the mean of three experimental values ± SEM. * $p < 0.05$, ** $p < 0.001$ when comparing the effect of a particular hypoxia with external normoxia. The dotted line represents the number of cells of inoculum.
